# Outbreak of Diarrhetic Shellfish Poisoning Associated with Mussels, British Columbia, Canada

**DOI:** 10.3390/md11051669

**Published:** 2013-05-21

**Authors:** Marsha Taylor, Lorraine McIntyre, Mark Ritson, Jason Stone, Roni Bronson, Olga Bitzikos, Wade Rourke, Eleni Galanis, Outbreak Investigation Team

**Affiliations:** 1 British Columbia Centre for Disease Control, 655 W 12 Ave, Vancouver, British Columbia, V5Z 4R4, Canada; E-Mails: lorraine.mcintyre@bccdc.ca (L.M.); eleni.galanis@bccdc.ca (E.G.); 2 Vancouver Coastal Health Authority, Vancouver, British Columbia, V5Z 4C2, Canada; E-Mails: mark.ritson@vch.ca (M.R.); olga.bitzikos@vch.ca (O.B.); 3 Fraser Health Authority, Suite 400, Central City Tower, 13450-102nd Avenue, Surrey, British Columbia, V3T 0H1, Canada; E-Mail: jason.stone@fraserhealth.ca; 4 Health Canada, C417, Frederick G Banting Bldg, Ottawa, Ontario, K1A 0K9, Canada; E-Mail: roni.bronson@hc-sc.gc.ca; 5 Dartmouth Laboratory, Canadian Food Inspection Agency, 1992 Agency Drive, Dartmouth, Nova Scotia, B3B 1Y9, Canada; E-Mail: wade.rourke@inspection.gc.ca; 6 School of Population and Public Health, University of British Columbia, 2206 East Mall, Vancouver, British Columbia, V6T 1Z3, Canada

**Keywords:** diarrhetic shellfish poisoning (DSP), outbreak, Canada, shellfish toxins, mussels, okadaic acid, public health

## Abstract

In 2011, a Diarrhetic Shellfish Poisoning (DSP) outbreak occurred in British Columbia (BC), Canada that was associated with cooked mussel consumption. This is the first reported DSP outbreak in BC. Investigation of ill individuals, traceback of product and laboratory testing for toxins were used in this investigation. Sixty-two illnesses were reported. Public health and food safety investigation identified a common food source and harvest area. Public health and regulatory agencies took actions to recall product and notify the public. Shellfish monitoring program changes were implemented after the outbreak. Improved response and understanding of toxin production will improve management of future DSP outbreaks.

## 1. Introduction

Diarrhetic Shellfish Poisoning (DSP) is caused by consuming sufficient amounts of okadaic acid (OA) group toxins, including OA, dinophysistoxin-1, and 2 (DTX-1, and DTX-2), which are produced by dinoflagellate algae (*Dinophysi*s spp., *Prorocentrum* spp*.*) [1,2], and dinophysistoxin-3 (DTX-3). DTX-3 refers to the fatty acid esters of OA, DTX-1 or DTX-2 and is found in phytoplankton and is also a product of shellfish metabolism [3,4,5]. The triggers for toxin production are unclear. Shellfish consume the algae and accumulate the heat stable, lipophilic toxins [6]. 

DSP is characterized by symptoms of nausea, vomiting, diarrhea, chills and abdominal pain [2]. The incubation period ranges from 30 min to 12 h and symptoms may last up to 3 days [7]. Chronic sequelae have not been reported, although there is very little known about possible health effects associated with chronic exposure to OA-group toxins or long term impact of acute intoxication [1,2,8]. The majority of DSP outbreaks have been reported outside of North America [2,6] but outbreaks and toxin detections (primarily DTX-1) have been reported from eastern Canada [9,10]. Although DTX-1 had been detected in the Northeastern Pacific marine sponge from British Columbia (BC) coastal waters, there were no confirmed reports of DSP prior to this outbreak [11]. Shellfish samples epidemiologically linked to illness are tested for lipophilic shellfish toxins (LSTs include OA-group toxins, pectenotoxins, azaspiracids, yessotoxins, and cyclic imine toxins), but clinical samples are not analysed for these toxins in Canada. 

OA-group toxins are monitored as part of the LSTs to meet Canadian Shellfish Sanitation Program requirements [12]. LSTs have been monitored in select BC sites since 2004 and the LST monitoring was expanded in 2011. Affected areas are closed to harvesting when action levels are exceeded [12]. As of July, 2011 the Health Canada (HC) action level for DSP toxins was 0.20 μg/g for the sum of OA and DTX-1 in edible shellfish tissue. DTX-2 and DTX-3 were not included in the action level prior to August, 2011. 

Monitoring DTX-3 directly in a regulatory laboratory is challenging because it may include many different compounds, but DTX-3 may be monitored indirectly by including either an enzymatic [13] or alkaline hydrolysis [14] step to remove the fatty acid esters and allow detection of the parent compound. The DTX-3 concentration is then calculated as the difference between the parent compound concentrations in the hydrolysed and unhydrolysed portions of the sample.

This paper describes a DSP outbreak in BC and outlines further areas of study to improve understanding and ability to investigate future outbreaks. 

## 2. Methods

Public health authorities collect clinical and exposure information using a standard surveillance form [15]. Surveillance for shellfish related illness is often based on clusters and not at the individual level. During this investigation, clusters reported to public health and the implicated food premises (restaurant or retail) were investigated to determine the source of the mussels and collect supplier information. A food safety traceback is conducted by tracking a food product from the retail level through distribution to the production or processing facilities to find the suspected source. The Canadian Food Inspection Agency (CFIA) conducted a traceback investigation using information collected from the food premises and provided HC with analytical results for OA-group toxins in mussel samples from the harvest area on August 5 and 6, 2011 for risk assessment.

The BC Public Health Microbiology and Reference Laboratory tested leftover food samples from implicated food premises for enteric pathogens and tested stool samples for norovirus and bacteria from individuals who were symptomatic and consumed the mussels. The CFIA tested shellfish samples from the implicated harvest area for LSTs using liquid chromatography-mass spectrometry (LC-MS/MS) [16], including an alkaline hydrolysis procedure to detect DTX-3 [14].

## 3. Results

On August 3, 2011, public health officials in two BC regional health authorities were notified of gastrointestinal illness among individuals who had consumed cooked mussels at different restaurants between July 28 and August 2. Information on symptoms, incubation period, and consumption of cooked products in various locations led to the hypothesis that DSP was the cause of illness. Restaurants receiving customer complaints notified the shellfish industry (harvester and processors) of the reported illnesses. The harvester took steps to withdraw product on August 3.

Sixty-two clinical cases associated with 15 food premises in three health authorities were reported. All cases reported consuming cooked mussels between July 28 and August 6. The most common symptoms were diarrhea, nausea, vomiting, abdominal pain and cramps. The incubation period ranged from 5 to 15 h and symptoms lasted 1 to 3 days.

**Figure 1:**
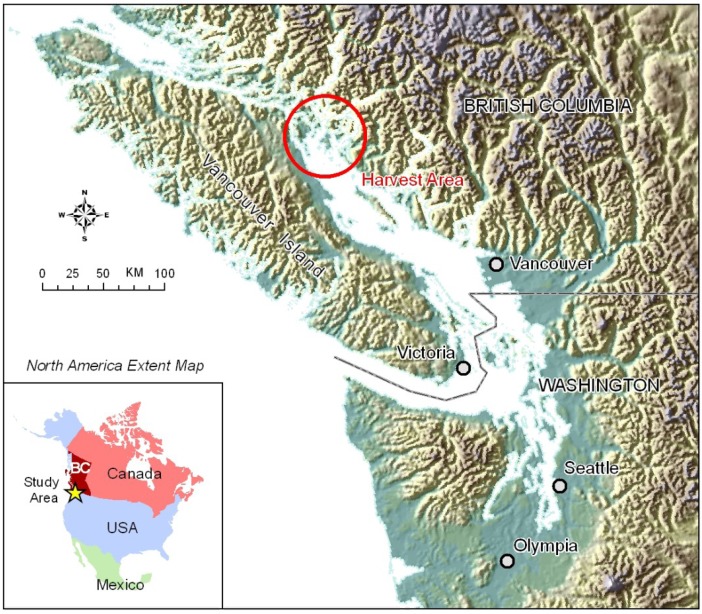
Location of harvest area associated with Diarrhetic Shellfish Poisoning (DSP) outbreak, British Columbia (BC), August 2011.

The food safety investigation and traceback identified a single harvest area at the north end of the Strait of Georgia (Figure 1) and a single mussel harvester. The harvest dates of implicated mussels were between July 24 and 31. OA-group toxin results of mussel samples from this harvest area are presented in Table 1. Two samples were reported above the action level of 0.2 μg/g for the sum of OA and DTX-1. Monitoring results demonstrated that concentrations of OA-group toxins in mussel samples were below the action level after August 8. Interestingly, DTX-3 was the most prominent toxin detected and was present in all mussel samples harvested after July 19. Two mussel samples and two sauces served with mussels at implicated restaurants tested negative for enteric pathogens. Six clinical stool samples were negative for norovirus and enteric pathogens.

**Table 1:** Okadaic acid (OA)-group toxin results from implicated harvest area, June–August 2011, BC, Canada.

Shellfish	Harvest date	Laboratory report date	OA (μg/g)	DTX-1 (μg/g)	DTX-3 (μg/g)
Mussel	June 21	June 28	ND	ND	ND
Mussel	July 5	July 14	ND	0.09	0.08
Mussel	July 12	July 20	ND	0.12	ND
Mussel	July 19	July 27	ND	0.18	0.23
Mussel	July 31	August 6	ND	0.21	0.26
Mussel	August 2	August 6	0.01	0.23	0.45
Mussel	August 2	August 6	ND	0.08	0.35
Mussel	August 2	August 6	ND	0.14	0.72
Mussel	August 4	August 5	ND	0.10	0.26
Mussel	August 8	August 13	ND	0.09	0.06
Mussel	August 17	August 24	ND	0.08	0.05

ND: Not detected above limit of quantitation (0.01 μg/g OA or 0.03 μg/g DTX-1); Note: DTX-2 was not identified in any analysed sample.

In addition to the harvester-led withdrawal of product on August 3, a harvest-area closure was recommended by the CFIA on August 5 based on OA-group toxin concentrations. On August 6, a health hazard alert recalling all implicated mussels harvested from the area was issued by the CFIA based on a HC risk assessment. The area was re-opened on August 24 following established procedures [10].

## 4. Discussion

Algal blooms leading to OA-group toxin production and shellfish contamination are increasing worldwide [2,6,17]. Although the causes for this increase are not clear, environmental factors such as climate change and projected increases to ocean temperatures, reduction in pH and increasing availability of nitrate may lead to increasing algal blooms. Increased marine traffic and global sales of spats for cultivation have been proposed as possible reasons for the emergence of toxic algal species in new areas. In addition, social factors such as an increased consumption of seafood, or improved regulatory standards and monitoring to identify toxins and human illnesses may also have impact on this apparent increasing trend [2,17,18]. The trigger for toxin production that led to this outbreak is unknown and further study in consultation with experts is needed. 

Washington State public health authorities also reported DSP cases in June, 2011 [19]. The cases also consumed mussels with high concentrations of DTX-1. Retrospectively, the similarity in timing and location of cases may suggest a common ecological reason for the emergence of OA-group toxins in Pacific Northwest coastal waters in 2011. Timely communication between departments and jurisdictions that share coastal waters will ensure that each is aware in the case of future events. 

There is international variation in OA-group toxin action levels and laboratory testing methods [1,6,20]. Regulatory testing most commonly employs LC-MS/MS or a mouse bioassay (to be suspended by the European Union after 2014). LC-MS/MS methods offer greater sensitivity and the ability to identify and quantify specific toxins and will be the primary method used internationally [1]. Canadian biotoxin monitoring is based on results of shellfish samples harvested from established sites. Some jurisdictions monitor phytoplankton levels in harvest-area water in addition to toxins in shellfish [6] and based on 2011 data from BC this approach was suggested by Esenkulova *et al.* to provide earlier warnings compared to biotoxin monitoring alone [21]. While some research has indicated a relationship between the increased concentration of phytoplankton and the toxin there have also been limitations noted [6,22,23]. The potential value of phytoplankton monitoring to compliment current LST monitoring in order to provide information on potential harmful algal blooms that may affect shellfish and human health and could lead to an earlier and reliable response in BC could be explored in collaboration with researchers and industry. 

A review of all HC marine biotoxin action levels was already in progress as of 2011, and an interim action level of 0.20 μg for the sum of OA, DTX-1, DTX-2 and DTX-3 per gram of edible shellfish tissue was provided by HC after reviewing LST results from this investigation [24]. Significant DTX-3 concentrations had not been detected in Canadian shellfish previously. Monitoring concentrations of DTX-3 is relevant because it may be present in higher concentrations than other OA-group toxins, as in our situation. In addition, there are indications that it can be hydrolysed to its parent compound in the gastrointestinal tract of humans [25]. Results from this DSP outbreak led to increasing the number of monitoring sites and sampling frequency in BC and the program has the capacity to expand and prioritize samples as necessary. Monitoring results are also provided to the shellfish industry as they become available [26]. These program changes in BC should further minimise the potential risk of contaminated shellfish entering commercial markets. Ongoing communication between jurisdictions and organizations as well as an understanding of factors contributing to OA-group toxin production should lead to improved identification and management in the event of a DSP outbreak. 

There is limited information on the epidemiology of human illness caused by OA-group toxins. Improvements in identification and surveillance of human illness, understanding of exposures, dose-response, severity of illness and chronic sequelae will improve our understanding of the burden of disease and ability to communicate on the risks associated with DSP, which may lead to better public health information and response. 

## 5. Conclusions

This DSP outbreak in BC was a significant event with 62 clinical illnesses reported and rapid action to identify and remove the source of illness. The intersectoral and collaborative approach to human illness surveillance and response to shellfish-related illnesses in BC led to rapid mitigation. Modifications to the monitoring program, which have already been implemented, will reduce the risk of DSP. Identifying factors contributing to toxin production could direct future monitoring plans. 
